# The distribution of hunger in Canadian youth

**DOI:** 10.24095/hpcdp.44.11/12.01

**Published:** 2024-11

**Authors:** Haleigh Cumiskey, Karen A. Patte, Valerie Michaelson, William Pickett

**Affiliations:** 1 Faculty of Applied Health Sciences, Brock University, St. Catharines, Ontario, Canada; 2 Department of Public Health Sciences, Queen’s University, Kingston, Ontario, Canada

**Keywords:** adolescent, epidemiology, hunger, food insecurity, pediatrics, youth

## Abstract

**Introduction:**

As a foundation for prevention, evidence is required to establish the contemporary distribution of hunger in Canadian adolescents. We present findings from a nationally representative survey of young Canadians on how perceived hunger is distributed demographically, socially and contextually.

**Methods:**

A probability-based sample of 15656 young Canadians aged 11 to 15 years who completed the 2017/18 cycle of the Health Behaviour in School-aged Children study was used. Descriptive statistics and multivariable regression analyses were used to profile the study population and the distribution of hunger attributed to “not having enough food at home.”

**Results:**

Overall, one in six (16.6%) survey participants reported experiencing hunger. There was a strong and significant correlation between low socioeconomic status and hunger (*p*<0.001 for the low and middle socioeconomic groups, compared to the high socioeconomic status group). Notably, 12.5% of participants with high levels of affluence also reported such experiences of hunger; however, this was not a statistically significant finding. Hunger was less frequently reported in older participants and in higher grade levels, with some level of significance. Regression analyses indicated that, within the sample, some demographic characteristics correlated with experiences of hunger: lower levels of affluence, identifying as male or nonbinary gender, long-term immigrant status, and identifying as Black, Latin American or mixed ethnicity.

**Conclusion:**

Clear disparities exist in the self-reported experience of hunger among young people in Canada.

**Highlights**

- Self-reported experience of hunger is a known indicator of social deprivation during childhood.
- One in six Canadian adolescents reported experiencing hunger due to a lack of food at home.
- At-risk groups included nonbinary, long-term immigrant, Black, Latin American and mixed ethnicity adolescents.
- Adolescents from affluent families sometimes reported hunger, suggesting that this indicator has different meanings to different groups of children.

## Introduction

Hunger and food insecurity are recognized public health priorities in Canada.^1^ They are complex issues that extend beyond the basic need to have reliable access to safe and adequate nutrition to the social and emotional circumstances within a young person’s environment.^2^ In 2021, 18.4% of Canadians lived in a food-insecure household^3^ and 16.8% of Canadians aged under 18 years lived in households experiencing moderate to severe food insecurity.^3^ Risk of experiencing food insecurity varies by sociodemographic factors; certain demographic, social and contextual factors may individually or cumulatively impact the likelihood that an individual is exposed to hunger at some point in their life.^1^,^4^ Children and adolescents appear to be at a disproportionately high risk relative to adult populations.^5^

Within Canadian adolescent populations, groups at an increased risk of experiencing food insecurity include those who identify as Black or Indigenous, those who come from single-guardian homes, and those who live in rented accommodation, in households where the highest level of education is secondary school and in households in which the guardian requires government social or disability-related supports.^6^

The impacts of hunger on the health and development of young people have been established.^7^,^8^ Adolescence represents a critical and sensitive period of the life course.^9^ Prolonged experiences of food insecurity and hunger can lead to an inability to meet certain “critical checkpoints”^10^ during this life stage, which may lead to negative health trajectories.^11^ Looked at in a more positive light, there is the real potential to impact hunger status if support is given at these critical points in time.^12^ Finding ways to better assist families and children who are deprived of life’s essentials will benefit populations from social, economic and health perspectives. Such initiatives are optimally based upon valid evidence describing patterns of hunger experienced by adolescent populations specifically, and not only descriptions of household food insecurity, because the two concepts, while highly related, are distinct. Yet, contemporary data on this public health issue are scarce in Canada. 

We had a unique opportunity to address this issue via an original analysis of nationally representative health survey data. Our goal was to describe and highlight various sociodemographic characteristic groups of Canadian youth aged 11 to 15 years who reported higher levels of hunger, as a basis for future prevention efforts and policy initiatives.

## Methods

***Study base***

The Health Behaviour in School-aged Children (HBSC) study is an ongoing, cross-national survey affiliated with the World Health Organization. Its protocol involves distribution of a standardized school-based survey every four years in up to 50 (mainly European) countries and regions.^13^ HBSC has been administered within Canada since 1989, with the eighth cycle administered in 2017/18.^14^ The survey protocol is available to the public.^15^ Available data include self-reported measures describing the health and well-being of adolescents aged 11 to 15 years. Response rates for the survey have been fairly consistent at approximately 74% each cycle.^13^

***Sample***

The 2017/18 Canadian survey involved 21 745 students from 287 schools in 10 provinces and 2 territories (Nunavut was unable to participate due to ethical principles associated with studying its highly Indigenous population). The initial sample of 21 745 participants was reduced to a final sample size of 15 656 in a complete case analysis, after removing individuals who did not meet the inclusion criteria (i.e. being aged 11–15 years; attending Grades 6–10; completing items core to this analysis). In addition, some exclusions related to the fact that some regions (Yukon, Northwest Territories, other local school boards) administered an abbreviated questionnaire in order to respect local levels of literacy or a lack of acceptance of specific survey topics. 

***Human subjects***

The HBSC study protocol holds ethics clearance from the Brock University Health Sciences Research Ethics Board (File No. 21-314), General Research Ethics Board at Queen’s University (TRAQ # 6010236), as well as the Health Canada-Public Health Agency of Canada Research Ethics Board (file number REB 2013-022P). 

***Key measures***

**Hunger**

A single questionnaire item asked participants to answer the following question: “Some young people go to school or to bed hungry because there is not enough food at home. How often does this happen to you?” Based on precedent, largely due to small cell sizes in more extreme categories (e.g. always), this item was dichotomized as those who had ever experienced hunger (responses of “sometimes,” “often” or “always”) versus those who had “never” experienced it.^16^

**Demographic measures**

Patterns of hunger were described within and across sociodemographic groups,^17^,^18^ i.e. age, grade level, gender, ethnicity, urban-rural geographic status, socioeconomic status and immigration status. “Age” and “grade level” were estimated by asking participants their birth month and year and comparing these with the date of survey administration, as well as what school grade they were currently enrolled in. The youngest group (participants aged 11) was assigned as the reference group. “Gender identity” was identified by asking participants “Are you male or female?” Response options included “male,” “female” and “neither term describes me” (interpreted as nonbinary gender). Males were assigned as the reference group. 

To determine “ethnicity,” 16 response options describing ethnicity, based upon a Statistics Canada classification,^19^ were grouped as follows into eight categories: White, Black, Latin American, Indigenous (First Nations, Mtis or Inuit), East and Southeast Asian (e.g. Cambodian, Indonesian), Indian and South Asian (e.g. Pakistani), Arab and West Asian (e.g. Afghan) and Other (including participants that selected multiple response options). Indigenous responses were suppressed in some analyses to adhere to ethics requirements. Participants within the largest group (those identifying as White) were assigned as the reference group. 

“Urban-rural geographic status” was defined based on the census subdivision where the school a participant attended was located, and varied from rural settings (<1000 persons and a population density of less than 400 persons per km^2^) to large urban population centres (100000+ persons per km^2^).^20^ Those within the most developed living centre (large urban population centre) were assigned as the reference group. 

“Perceived socioeconomic status” (i.e. affluence) was determined by asking the following question: “How well off do you think your family is?” Responses were categorized into three groups based on precedent:^21^ low (“not very well off” and “not at all well off”), middle (“average”) and high (“very well off” and “quite well off”). Those in the group with the highest socioeconomic status were assigned as the reference group. 

“Immigration status” was determined by asking the following questions: “In which country were you born?” Response options were “Canada,” “Other (please specify)” and “I don’t know.” Participants were then asked, “If you were not born in Canada, how many years have you lived in Canada?” Five possible response options were collapsed into three groups, as per precedent:^22^ born in Canada, recent immigrants (1–5 years) and long-term immigrants (>5 years). Those born in Canada, the largest group, were assigned as the reference group. 

***Statistical analyses***

The sample was profiled by sociodemographic characteristics. Experiences of hunger were first described in a bivariate manner according to available sociodemographic factors. We then explored variations in hunger via multivariable negative binomial regression models that examined hunger as a function of all key sociodemographic variables, with simultaneous control for all available variables (i.e. age, gender, ethnicity, urban-rural geographic status, socioeconomic status and immigration status) to account for mutual confounding. Adjusted prevalence ratios were presented as estimates of relative risk, consistent with the cross-sectional nature of the data. All analyses were performed in SPSS version 29,^23^ with the level of statistical significance for correlations set at *p*<0.05. Confidence intervals were generated based on model estimates and available sample size, by multiplying the standard error around the estimates by 1.96, with an adjustment for clustering at the school level by including a school code as a random effect. The data were also weighted to ensure national representation. 

Given the importance of considering intersecting social positions, we conducted exploratory analyses investigating the connection between socioeconomic status, gender and reports of hunger. Confidence intervals were generated around each prevalence estimate using the same methodology as the multivariable regression analyses. 

## Results

The available sample is described in Table 1. As per the recruitment strategy, there were five age groups, each with roughly the same number of participants, and five grade level groups, also with roughly the same number of participants in each. There were slightly fewer males than females, while self-identified nonbinary participants made up a very small proportion of the sample (1.2%). Most participants identified as having a White (71.2%) or Other or mixed (12.1%) ethnic identity. Most participants attended schools within a small (44.6%) or a large (36.2%) population centre. Finally, most participants were born in Canada (75.5%) or were long-term immigrants (19.5%). 

**Table 1:** Demographic characteristics of the study sample, 2017/18 Health Behaviour in School-aged Children study, Canada

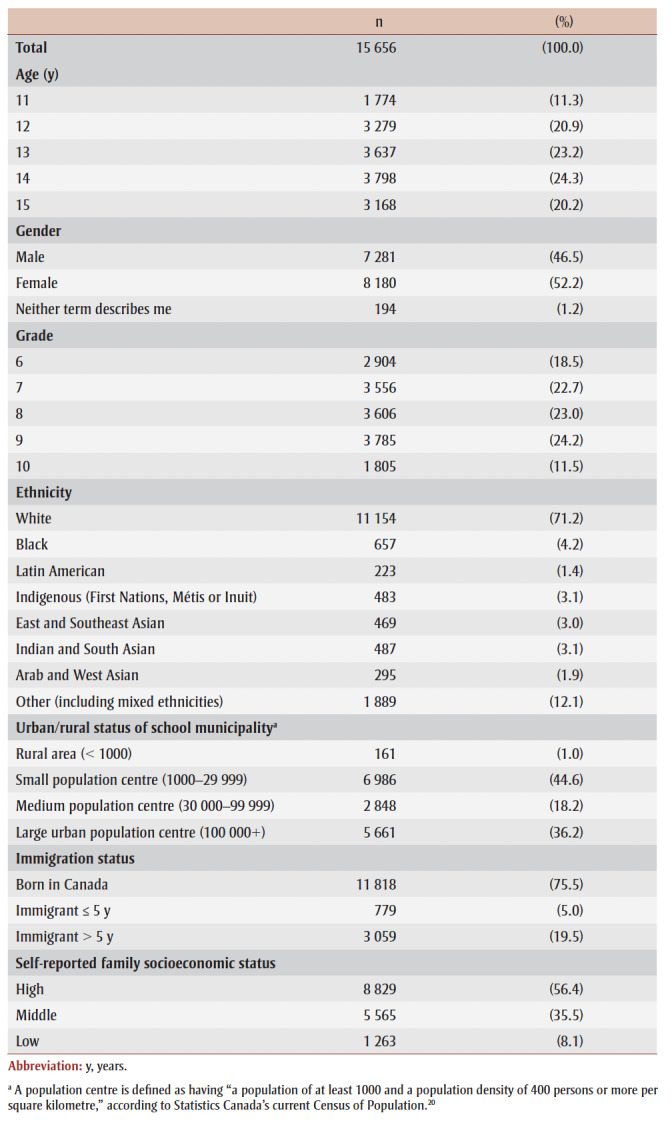

***Hunger and its patterns***

Variations in hunger were described by sociodemographic factors, including the results of the fully adjusted negative binomial regression models (Table 2). Compared to the youngest participants, the oldest two groups (those aged 14 and 15years), were significantly less likely to experience hunger. Males were significantly more likely than females to experience hunger. Those who identified as nonbinary appeared to be disproportionately at higher risk, although this finding was not statistically significant (*p*=0.07).

**Table 2:** Self-reported experience of hunger and its correlation with sociodemographic indicators, 2017/18 Health Behaviour in School-aged Children study, Canada

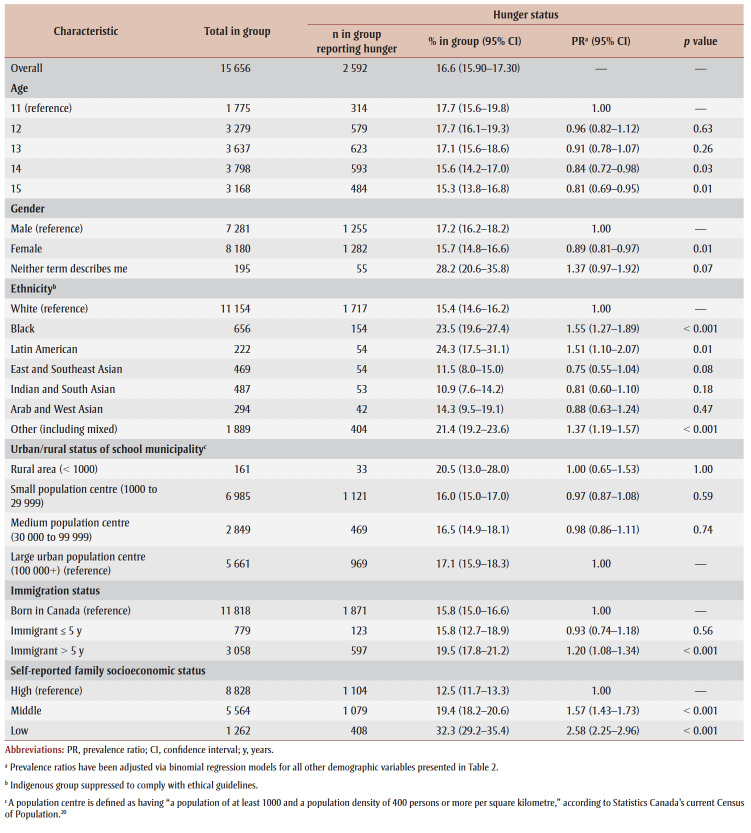

Several ethnic groups were at a higher risk of experiencing hunger. Compared to those who identified as White, participants identifying as Black, Latin American and Other or mixed reported the highest levels. Participants who attended schools in rural areas were the most likely to experience hunger (20.5%), followed by those at schools in a large urban setting (17.1%). However, there were no statistically significant differences in the risk of reporting experiences of hunger by population centre size. Long-term immigrants were significantly more likely to experience hunger compared with those born in Canada (19.5% vs. 15.8%, respectively; prevalence ratio [PR]=1.20, *p*<0.001). 

As expected, the strongest correlation was observed with the measure of socioeconomic status; participants classified in the low socioeconomic group reported hunger 2.6 times as frequently as those in the high socioeconomic group (32.3% in low, 12.5% in high). This correlation was significant in both the middle and low groups (PR=1.57 [*p* <0.001] and PR=2.58 [*p*<0.001], respectively), compared to high. 

**Figure 1:**
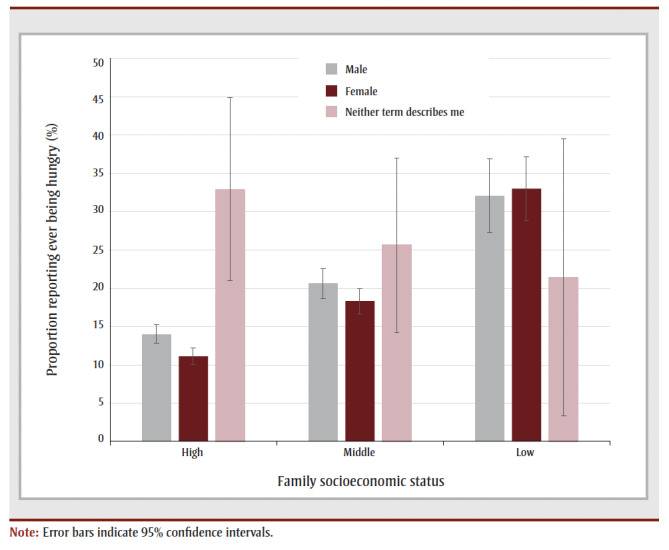
Frequency of hunger among Canadian youth, stratified by gender identity and socioeconomic status, 2017/18 Health Behaviour in School-aged Children study, Canada

Figure 1 presents the frequency of self-reported hunger stratified by socioeconomic group and gender. For males and females, as socioeconomic status decreased, the proportion within each gender group increased. In contrast, of all nonbinary participants affected by hunger, the largest proportion within this group (33%) were within the high socioeconomic group. 

## Discussion

This novel analysis examined experiences of hunger in a large, nationally representative sample of young Canadians, and profiled these experiences of hunger from a sociodemographic perspective. Rather than explaining the underlying reasons for observed variations, our goal was to identify important variations in experiences of hunger to inform both etiological research and eventual prevention efforts. The most important finding was that approximately one in six young Canadians aged 11 to 15years reported that they experienced some level of hunger due to not having enough food at home. The strongest observed pattern within our analyses was the correlation between hunger with lower socioeconomic status. Nonbinary gender participants were disproportionately affected by hunger compared to participants who identified as male or female, providing further indication of the social stratification of hunger experiences by gender. Additional sociodemographic groups at higher risk for experiencing hunger included those who identified as male, Black, Latin American or Other or mixed ethnicity, those who were long-term immigrants, and those who attended schools in rural areas. 

The relationship of hunger with socioeconomic status, while not unexpected,^18^ is a particularly important finding. The questionnaire item used to establish experiences of hunger was introduced originally to the HBSC study as a measure of extreme deprivation,^16^ as socioeconomic status has been closely linked with hunger in various adult, child and adolescent populations.^17^,^24^ Our findings show that prevalence levels of self-reported hunger were highest in the lowest socioeconomic group, consistent with this past evidence.^25^

Interestingly, experiences of hunger were also reported by over one-tenth of young Canadians who reported having above average wealth. This finding suggests that the measure of hunger may have different meanings in different socioeconomic contexts, and with other factors (e.g. a lack of organization in the home^2^) potentially determining perceptions of hunger, even in the presence of affluence. To illustrate, some families may have the means to purchase food, but they may not do so reliably.^2^ Alternatively, this finding may reflect an expression of privilege bordering on entitlement;^26^ there may be sufficient food in the home to satisfy nutritional needs, but the food may not fit with their taste or other preferences, and so, adolescents may opt to go hungry.^27^

Correlations between hunger and other sociodemographic factors were also identified. Males reported hunger marginally more often than females. This is unusual, as females typically report higher frequencies of food insecurity.^28^ This result may have biological explanations in relation to sex differences in average nutrition needs; adolescent males require approximately 500 additional calories per day compared to females.^29^ Alternatively, it may be attributable to the greater social acceptance of various forms of restrictive eating and dieting among girls than boys, given gendered differences in sociocultural appearance ideals.^30^ More striking was the potential association of higher levels of hunger and identifying as nonbinary, which may be reflective of cumulative disadvantage among this at-risk group.^31^ Nonbinary youth are more likely to experience personal body dissatisfaction and low self esteem, and may also experience body dysmorphia.^32^,^33^ This may lead to a disordered relationship with food and be partially responsible for this study’s results. 

Ethnicity was also correlated with hunger. Those who identified as Black, Latin American or Other or mixed ethnicity were at the highest risk of hunger, which is not uncommon among Canadian census studies.^34^ Interestingly, some ethnic groups (East and Southeast Asian, Indian and South Asian, Arab and West Asian) were at a lower risk compared to White participants. This may be due to a variety of factors, including the presence of cultural food systems, household family structure or community ties.^34^,^35^ Such hypotheses warrant focussed investigation. Similarly, relationships between hunger and immigration status are provocative. Consistent with the “healthy immigrant effect,”36 after coming to Canada there is often a period when immigrants have better overall health compared to their native-born counterparts.^35^ New immigrants may have access to resources and support that foster their assimilation in Canada, while long-term immigrants may experience various forms of hardship as they continue to live in the country, increasing the potential for disparities such as disproportionate hunger and food insecurity.^36^

Patterns of hunger by gender and socioeconomic status were also unexpected. The nonbinary participants who reported experiencing hunger most frequently were those who were part of the highest socioeconomic group. While unexpected, this finding demonstrates that the social roots of hunger do not always relate to poverty. Perhaps there are other hypotheses and pathways at work that underlie this pattern, such as the need for young people with nonbinary identities to conform with diets and lifestyles that undermine their health.^32^ Potential misclassification by self-report may be responsible for some of this observation: a proportion of respondents may report that their family is “quite well off” when in reality they have faced financial struggle. This would explain the same respondent noting that they were in fact experiencing hunger due to a lack of food in the home. 

***Strengths and limitations***

The strengths and limitations of this study warrant comment. In terms of strengths, first, the analysis highlights that hunger in children and youth is a topic of national importance and remains an endemic issue in our country.^37^ Second, we profiled variations in hunger and identified several high-risk groups in a focussed equity analysis that included both bivariate and multivariable analyses. Third, the analysis benefited from the existence of an established cross-national research protocol with validated and well tested items and a robust national sample. 

With respect to the limitations, first, because some jurisdictions shortened the questionnaire to respect local levels of literacy and cultural sensitivities, the effective sample size was reduced for this analysis. This may have also impacted groups who are often considered equity-denied populations,^38^ and therefore has reduced the diversity and inclusivity of the sample. Second, due to privacy concerns, modelling results regarding Indigenous participants were suppressed to adhere to ethical research guidelines. Again, this may have impacted the inclusivity of the study sample. 

Third, as the HBSC is a cross-sectional study, temporality cannot be inferred from many analyses, limiting the potential for causal inference. Hence, all effects that were estimated should be considered correlational. Fourth, prevalence estimates of hunger may be biased downward due to nonparticipation in the survey by at-risk children. The effects of this pattern of nonresponse on the sociodemographic patterns of hunger remain unknown, although we speculate it is likely that any effect of this nonparticipation would be to bias the results toward the null.

This study was able to highlight high risk groups of young Canadians who are more likely to experience hunger. While family income and hunger were highly correlated, access to nutrition may extend beyond income to other contextual factors. Future research on youth hunger and food insecurity may be guided by the goal of describing some of the complex interactions between the various demographic and social characteristics highlighted here that may lead to a young person experiencing hunger. Additionally, the results of this study may be beneficial to other research groups looking to develop hypotheses regarding health equity on a larger scale, as it is clear that there are systemic discrepancies in the ways various groups of people access basic resources such as adequate nutrition. 

## Conclusion

In this brief report, we have profiled experiences of hunger among young Canadians. Hunger is experienced in varying frequencies among different sociodemographic groups. The results of this analysis provide insight into hunger and its potential determinants, and foster hypotheses that support both etiological and interventional research in this important social field. 

## Acknowledgements

Health Behaviour in School-aged Children (HBSC) is an international study carried out in collaboration with WHO/EURO. The HBSC study is funded in Canada by the Public Health Agency of Canada (6D016-204692/001/SS), and the analysis was supported with graduate funding by Brock University. The international coordinator of the 2018 survey was Dr. Jo Inchley (Glasgow University, Scotland) and the data bank manager was Dr. Oddrun Samdal (University of Bergen, Norway). Principal investigators are Dr. Wendy Craig (Queen’s University) and Dr. William Pickett (Brock University and Queen’s University), and its national coordinator is Mr. Matthew King (Queen’s University). Karen A. Patte is the Canada Research Chair in Child Health Equity and Inclusion and has contributed to the report through revisions and development of the final manuscript. 

## Conflicts of interest

The authors declare no conflicts of interest.

## Authors’ contributions and statement

HC: conceptualization, formal analysis, project administration, writing—original draft.

VM, KP: conceptualization, supervision, writing—review and editing.

WP: conceptualization, data curation, funding acquisition, project administration, supervision, writing—review and editing.

The content and views expressed in this article are those of the authors and do not necessarily reflect those of the Government of Canada.

## References

[B01] Household food insecurity, 2017/2018 [Internet]. Government of Canada.

[B02] Pickett W, Michaelson V, Davison C (2015). Beyond nutrition: hunger and its impact on the health of young Canadians. Int J Public Health.

[B03] Table 13-10-0835- 01: Food insecurity by selected demographic characteristics [Internet]. Statistics Canada.

[B04] HungerCount 2023 [Internet]. Food Banks Canada.

[B05] Bhawra J, Kirkpatrick SI, Hammond D (2021). Food insecurity among Canadian youth and young adults: insights from the Canada Food Study. Can J Public Health.

[B06] Liu R, Urquia ML, Tarasuk V (2023). The prevalence and predictors of household food insecurity among adolescents in Canada. Can J Public Health.

[B07] Ke J, Ford-Jones EL (2015). Food insecurity and hunger: a review of the effects on children’s health and behaviour. Paediatr Child Health.

[B08] Kirkpatrick SI, McIntyre L, Potestio ML (2010). Child hunger and long-term adverse consequences for health. Arch Pediatr Adolesc Med.

[B09] Johnson MK, Crosnoe R, GH Jr (2011). Insights on adolescence from a life course perspective. J Res Adolesc.

[B10] Haas S (2008). Trajectories of functional health: the “long arm” of childhood health and socioeconomic factors. Soc Sci Med.

[B11] Barban N (2013). Family trajectories and health: a life course perspective. Eur J Popul.

[B12] Hargreaves D, Mates E, Menon P, et al (2022). Strategies and interventions for healthy adolescent growth, nutrition, and development. Lancet.

[B13] Health Behaviour in School-aged Children (HBSC) study [Internet]. WHO.

[B14] Craig W, Pickett W, King M The health of Canadian youth: findings from the Health Behaviour in School-aged Children Study. Public Health Agency of Canada.

[B15] Roberts C, Currie C, Samdal O, Currie D, Smith R, Maes L (2007). Measuring the health and health behaviours of adolescents through cross-national survey research: recent developments in the Health Behaviour in School-aged Children (HBSC) study. J Public Health.

[B16] Griebler R, Molcho M, Samdal O, et al (2009). Health Behaviour in School-Aged Children: a World Health Organization cross-national study. LBPHIR and Edinburgh (UK): CAHRU.

[B17] Kirkpatrick SI, Tarasuk V (2008). Food insecurity in Canada. Can J Public Health.

[B18] Household food insecurity in Canada [Internet]. PROOF.

[B19] Ethnic or cultural origins: technical report on changes for the 2021 Census [Internet]. Government of Canada.

[B20] Dictionary, Census of Population, 2016: population centre [Internet]. Government of Canada.

[B21] Currie CE, Elton RA, Todd J, Platt S (1997). Indicators of socioeconomic status for adolescents: the WHO Health Behaviour in School-aged Children Survey. Health Educ Res.

[B22] Davison CM, Torunian M, Walsh P, Thompson W, Pickett W (2013). Bicycle helmet use and bicycling-related injury among young Canadians: an equity analysis. Int J Equity Health.

[B23] (2023). IBM SPSS statistics for Windows, version 29.0. IBM.

[B24] Orgut I, III LG, Davis LB, et al, Zobel CW, Altay N, Haselkorn MP (2016). Achieving equity, effectiveness, and efficiency in food bank operations: strategies for feeding America with implications for global hunger relief. Springer International.

[B25] Kansanga MM (2022). ‘Hunger in early life’: exploring the prevalence and correlates of child food insecurity in Canada. Agric Food Secur.

[B26] Luthar SS (2003). The culture of affluence: psychological costs of material wealth. Child Dev.

[B27] Viljakainen J, Figueiredo RA, Viljakainen H, Roos E, Weiderpass E, Rounge TB (2019). Eating habits and weight status in Finnish adolescents. Public Health Nutr.

[B28] Broussard NH (2019). What explains gender differences in food insecurity. Food Policy.

[B29] Soliman A, Sanctis V, Elalaily R (2014). Nutrition and pubertal development. Indian J Endocrinol Metab.

[B30] Culbert KM, Sisk CL, Klump KL (2021). A narrative review of sex differences in eating disorders: is there a biological basis. Clin Ther.

[B31] Russomanno J, Tree JM (2020). Food insecurity and food pantry use among transgender and gender non-conforming people in the Southeast United States. BMC Public Health.

[B32] Bell K, Rieger E, Hirsch JK (2019). Eating disorder symptoms and proneness in gay men, lesbian women, and transgender and gender non-conforming adults: comparative levels and a proposed mediational model. Front Psychol.

[B33] Cusack C, Iampieri A, Galupo M (2022). “I’m still not sure if the eating disorder is a result of gender dysphoria”: trans and nonbinary individuals’ descriptions of their eating and body concerns in relation to their gender. Psychol Sex Orientat Gend Divers.

[B34] Table 13-10-0835- 01: Food insecurity by selected demographic characteristics [Internet]. Government of Canada.

[B35] Greenwald HP, Zajfen V (2017). Food insecurity and food resource utilization in an urban immigrant community. J Immigr Minor Health.

[B36] Ichou M, Wallace M (2019). The healthy immigrant effect: the role of educational selectivity in the good health of migrants. Demogr Res.

[B37] (2023). Priority actions for immediate acceleration in response to the global food and nutrition crisis [Internet]. Food and Agriculture Organization of the United Nations.

[B38] Bauer GR (2014). Incorporating intersectionality theory into population health research methodology: challenges and the potential to advance health equity. Soc Sci Med.

